# Irrational and Inappropriate Use of Antifungals in the NICU: A Narrative Review

**DOI:** 10.3390/antibiotics15010073

**Published:** 2026-01-09

**Authors:** Niki Dermitzaki, Foteini Balomenou, Chrysoula Kosmeri, Maria Baltogianni, Aikaterini Nikolaou, Anastasios Serbis, Vasileios Giapros

**Affiliations:** 1 Neonatal Intensive Care Unit, School of Medicine, University of Ioannina, 45500 Ioannina, Greece; n.dermitzaki@uoi.gr (N.D.); f.balomenou@uoi.gr (F.B.); mbalt@doctors.org.uk (M.B.); nikaikaterini@gmail.com (A.N.); 2 Pediatric Department, School of Medicine, University of Ioannina, 45500 Ioannina, Greece; chrisa.kosmeri@gmail.com (C.K.); aserbis@uoi.gr (A.S.)

**Keywords:** neonatal candidiasis, antifungal drugs, antifungal prophylaxis, *Candida* resistance, antifungal stewardship

## Abstract

Invasive *Candida* infections in the neonatal intensive care unit (NICU) are associated with significant morbidity and mortality, particularly among extremely preterm neonates. Early treatment with antifungals is critical to improve survival rates and avoid long-term adverse outcomes. Prevention with antifungal prophylaxis in high-risk neonates has been shown to reduce the prevalence of invasive *Candida* infections effectively. However, the irrational and/or inappropriate use of antifungals has been documented. This narrative review aims to provide an overview of the rationales for the inappropriate use of antifungals in the NICU, the consequences that ensue, and the promising strategy of antifungal stewardship programs to optimize antifungal use. The nonspecific clinical presentation of systemic *Candida* infections and the lack of rapid, accurate diagnostic techniques for *Candida* identification and specification in most settings lead to a high rate of empirical treatment in neonates without a proven infection. Moreover, evidence on the optimal dosing of antifungal agents and the treatment duration in the neonatal population is lacking, which may result in excessive or subtherapeutic drug exposure. Antifungal misuse is associated with microbiological consequences, including the emergence of antifungal-resistant *Candida* strains, and clinical consequences, such as drug toxicities and alterations in the intestinal mycobiome. It is therefore imperative to optimize antifungal use in the NICU. The implementation of antifungal stewardship programs, which, through a multidisciplinary approach, aim to improve diagnosis and guide clinicians on antifungal selection, dosing, and duration for both prevention and treatment according to the local epidemiology, represents a promising strategy for antifungal optimization in the NICU.

## 1. Introduction

### 1.1. Epidemiology

Neonatal invasive infections are a major cause of morbidity and mortality in Neonatal Intensive Care Units (NICUs). The causative pathogens implicated in neonatal sepsis vary significantly depending on the time of infection, the gestational age (GA) and the geographical region [1]. The most prevalent pathogens observed in NICUs are Gram-positive bacteria, such as *Staphylococcus aureus*, *coagulase-negative Staphylococci*, and *Streptococcus* spp., Gram-negative bacteria, including *Enterobacterales*, predominantly *Escherichia coli* and *Klebsiella pneumoniae*, and fungi, most commonly *Candida* spp. [1,2,3].

*Candida* infections represent a significant concern in NICUs worldwide. The estimated prevalence among admitted neonates is 0.5–2% [4]. The risk of invasive candidiasis has a reverse correlation with both GA and birthweight (BW), and is reported to affect 4–8% of extremely low birthweight (ELBW) neonates [5]. A multicenter study from the US reported that *Candida* spp. was isolated in 5.1% of late-onset sepsis (LOS) episodes in neonates with a GA of less than 29 weeks. However, in the subgroup of neonates with a GA less than 23 weeks, 10% of LOS episodes were attributed to *Candida* spp. [3]. Neonatal candidiasis is associated with significant morbidity and mortality. The mortality rate has been reported to range from 14 to 50%, with the highest rates observed among the most vulnerable population of very low birth weight (VLBW) neonates [6,7]. Survivors are at an increased risk of severe neurodevelopmental impairment [6,8,9,10].

The incidence of neonatal candidiasis varies significantly across different geographic regions. Considerably higher prevalence rates are observed in low- and middle-income countries than in high-income countries [4,11]. Interestingly, it has been observed that neonates affected in low- and middle-income countries are more mature than those affected in high-income countries [4,11,12]. A recent systematic review reported a mean GA of 31.4 weeks for neonates with invasive candidiasis in low- and middle-income countries, whereas the median GA in a multinational European study was 27 weeks [4,13]. This observation is probably attributable to the limited survival of very preterm neonates in low- and middle-income countries, a substantial proportion of whom do not survive long enough to be exposed to the predisposing factors of *Candida* infections. However, the high rate of horizontal healthcare transmission, due to limitations in infection control practices, primary prevention, and the widespread use of broad-spectrum antibiotics, contributes to high infection rates among more mature neonates who survive [4,11].

*Candida albicans* represents the most prevalent strain responsible for neonatal invasive infections [5,9,12]. However, considerable heterogeneity is evident among geographic regions concerning species distribution and susceptibility patterns [14]. It has been documented that the prevalence of non-*albicans* strains is increasing worldwide, particularly in low- and middle-income countries [15]. Among non-*albicans Candida* spp. the most frequently reported are *Candida parapsilosis*, *Candida glabrata*, *Candida tropicalis*, and *Candida auris* [7,11,12,13,16,17]. In addition to *Candida* spp., other fungi, including *Aspergillus*, *Malassezia*, and *Cryptococcus*, have been reported infrequently as causative agents of neonatal invasive infections [18,19].

### 1.2. Clinical Presentation

The clinical presentation of invasive candidiasis in neonates is non-specific and may resemble that of bacterial sepsis, with apnea, cardiopulmonary instability, lethargy, feeding intolerance, temperature instability, and other sepsis-like symptoms and signs [20]. Dissemination and deep-tissue infection may occur. The most prevalent sites of infection are the central nervous system (CNS), kidneys, heart, eyes, liver, and spleen [21,22]. The usefulness of available diagnostic techniques is restricted by the reduced sensitivity and slow turnaround time of traditional methods, as well as the limited availability of recently evolved molecular techniques [23,24]. The non-specific clinical presentation and the significant limitations of diagnostic methods render the diagnosis of systemic candidiasis challenging.

### 1.3. Antifungal Prophylaxis

In addition to prematurity and low birth weight, several other predisposing factors for invasive *Candida* infections have been recognized in hospitalized neonates. These include broad-spectrum antibiotics, particularly third-generation cephalosporins and carbapenems; central venous catheter placement and other invasive procedures; total parenteral nutrition; corticosteroids; and gastrointestinal pathologies [25,26,27,28,29]. Antifungal prophylaxis is administered to preterm neonates at a greater risk for invasive *Candida* infections [26]. Fluconazole is the agent of choice for antifungal prophylaxis [30]. The Infectious Diseases of North America (IDSA) and the European Society of Clinical Microbiology and Infectious Diseases (ESCMID) guidelines recommend fluconazole prophylaxis for ELBW in settings with a high incidence of invasive candidiasis (>10%). For NICUs with a lower incidence of candidiasis, an individualized approach is recommended for ELBW, with risk stratification based on the presence of additional risk factors [31,32].

### 1.4. Overview of Antifungals Used in Neonates

In the treatment of neonatal invasive *Candida* infections, four classes of antifungals are employed (Table 1). Polyenes, particularly amphotericin B deoxycholate (D-Amb), are the most frequently used drugs [33]. Amphotericin is generally effective against *Candida albicans* and various non-*albicans* species (e.g., *Candida parapsilosis*, *Candida tropicalis*, *Candida krusei*), but not against *Candida lusitensis* and *Candida auris* [34]. The nephrotoxicity associated with amphotericin, which is well-documented in both pediatric and adult populations, is less frequent in neonates, probably due to different pharmacokinetic parameters. In addition, other potential adverse effects, such as hepatotoxicity and hypokalemia, are less common in neonates [33,35,36]. To mitigate the risk of adverse effects, lipid formulations of amphotericin (L-Amb) have been developed. However, in neonates, D-Amb is usually preferred due to better renal penetration [31,37]. All amphotericin formulations are administered intravenously [38]. The IDSA and the ESCMID guidelines both recommend D-Amb as the primary treatment for neonatal candidiasis, with fluconazole as an option for neonates who have not previously received fluconazole prophylaxis [31,32].

Fluconazole is a first-class triazole, widely used in NICUs. It is effective against various *Candida* species, except *Candida krusei*, *Candida glabrata*, and *Candida auris* [18]. However, the emergence of resistant strains is becoming increasingly documented [39]. Fluconazole has excellent oral bioavailability and a favorable safety profile in neonates. Hepatotoxicity has been reported, and the inhibition of cytochrome P450 may lead to interactions with other drugs [40]. Other azoles, such as itraconazole and voriconazole, are not approved for use in neonates due to limited pharmacokinetic and safety data. The broader spectrum of activity of voriconazole, including *Candida krusei*, *Candida glabrata*, and *Candida auris*, has led to its use in refractory cases as a salvage therapy [32,33,41].

The echinocandins, micafungin, caspofungin, and anidulafungin are effective against various *Candida* strains, including *Candida albicans* and various non-*albicans* species, such as *Candida glabrata*, *Candida krusei*, *Candida auris*, and *Candida lusitaniae* [42,43,44]. Echinocandins have been demonstrated to have a favorable safety profile in the neonatal population and a minimal propensity to interact with other drugs [45,46,47,48]. However, the available literature is limited, and micafungin is currently the only echinocandin approved for use in neonates [49,50]. The broad range of activity, in addition to the activity against biofilms, renders them a reasonable choice in neonatal *Candida* infections. Indeed, both the IDSA and the ESCMID guidelines recommend the use of micafungin or caspofungin in cases of resistance or toxicity to conventional antifungals [31,32].

Flucytosine, a nucleoside analog, is recommended in combination with amphotericin in cases of difficult-to-treat CNS infections [31]. The rapid emergence of resistance precludes its use as monotherapy [51]. Flucytosine has been associated with various adverse effects, including nephrotoxicity, hepatotoxicity, and bone marrow suppression [52].

Prompt initiation of appropriate antifungal treatment is critical for the survival and long-term outcomes of affected neonates [38]. However, the non-specific clinical presentation and the lack of rapid, accurate diagnostic techniques for *Candida* in most settings often lead to the initiation of empirical antifungal treatment. Indeed, several studies have demonstrated that a small proportion of neonates treated with antifungals have proven *Candida* infection [53,54]. Moreover, antifungal prophylaxis is often administered in infants without a clear indication [53]. The irrational and inappropriate use of antifungals has been demonstrated to be associated with the emergence of resistance and an increased risk of drug toxicity [55]. Due to the rarity of non-*Candida* fungal infections in neonatal populations, they are not common targets of antifungal prophylaxis, and they do not represent a significant driver of irrational or inappropriate antifungal use in the NICU [18,19]. This narrative review aims to summarize the antifungal use in the neonatal population, with a focus on the rationales for inappropriate use and the consequences that ensue. Secondly, the aim is to provide an overview of antifungal stewardship programs to optimize antifungal therapy in this vulnerable population.

**Table 1:** Mechanism of action and adverse effects of antifungal drugs.

Antifungal Class	Antifungal Agent	Mechanism of Action	Approved for Use in Neonates	Adverse Effects
Polyenes [40,56]	Amphotericin B deoxycholate	Binding to ergosterol (component cytoplasmic membrane), leading to pore formation and increased permeability leading to cell death	Yes	Nephrotoxicity Electrolyte disturbances
	Liposomal amphotericin B		Yes	Less adverse effects compared to Amphotericin B deoxycholate
Triazoles [39,40,57]	Fluconazole	Disruption of ergosterol biosynthesis, by inhibition of 14-a-sterol demethylase (cytochrome P-450 enzyme)	Yes	Hepatotoxicity Nephrotoxicity Gastrointestinal irritation QT prolongation Drug–drug interactions
	Itraconazole		No	Hepatotoxicity Nephrotoxicity Cardiac effects Gastrointestinal irritation Drug–drug interactions
	Voriconazole		No	Hepatotoxicity Visual disturbances Photosensitivity QT prolongation Drug–drug interactions
Echinocandins [58]	Micafungin	Disruption of synthesis of 1,3-beta-D-glucan (component of the fungal cell wall, essential for structural integrity)	Yes	Hepatotoxicity Gatsrointestinal irritation Hypokalemia
	Caspofungin		No	Hepatotoxicity Gatsrointestinal irritation Hypokalemia
	Anidulafungin		No	Hepatotoxicity Gatsrointestinal irritation Hypokalemia Potential accumulation of polysorbate 80
Nucleoside analogue [52]	Flucytosine	Disruption of RNA and inhibition of DNA synthesis in the fungal cell	Yes	Hepatotoxicity Nephrotoxicity Bone marrow suppression Gastrointestinal irritation

RNA: ribonucleic acid; DNA: deoxyribonucleic acid.

## 2. Methods

To identify relevant literature, a structured and comprehensive search was conducted using online databases: PubMed, Scopus, and Google Scholar from inception up to September 2025, using the following keywords: neonatal candidiasis; antifungal drugs; antifungal prophylaxis; *Candida* resistance; antifungal stewardship. Only full-text, peer-reviewed studies written in English were included. Articles not related to neonatal *Candida* infections and treatment, or involving non-*Candida* fungal infections, were excluded. The reference lists of the retrieved articles were reviewed to assess for relevant studies that may not have been detected in the initial search. The article selection process and the subsequent data extraction were conducted independently by two authors. This process involved screening the titles and abstracts of articles, followed by a full-text evaluation. In cases of uncertainty, the decision-making process was conducted through discussions with the coauthors.

The initial database research retrieved approximately 900 records. Following the removal of duplicates, approximately 640 records were screened by title and abstract, and 430 records were not relevant to neonatal candidiasis, antifungal prophylaxis, or treatment and were excluded. The full texts of 210 articles were assessed for eligibility; 115 were excluded due to a lack of relevance. Ultimately, 159 references were used in this narrative review, including 64 studies that were retrieved manually from the reference lists of the included articles.

## 3. Factors Contributing to Inappropriate Use of Antifungals in Neonates

### 3.1. Challenges in Establishing a Definitive Diagnosis

The clinical diagnosis of systemic *Candida* infections in neonates is particularly challenging due to the nonspecific clinical presentation, which is indistinguishable from that of bacterial sepsis. Consequently, in suspected cases, the diagnosis depends on the diagnostic techniques. However, the available diagnostic methods have substantial limitations.

The isolation of *Candida* spp. in blood cultures is regarded as the gold standard for diagnosis [24]. However, the sensitivity of blood cultures is low, with a reported range of up to 50%, and is contingent on the volume of blood obtained [24,59,60]. Moreover, the long turnaround time between 24 and 72 h delays diagnosis [61]. The serum biomarker 1,3-β-D-glucan is useful for excluding *Candida* infection, as it has a high negative predictive value but is associated with a high false-positive rate. The optimal positivity threshold in neonates is yet to be determined [62,63]. Mannan/anti-mannan antibodies, serum biomarkers specific for *Candida* diagnosis, are characterized by early positivity, rapid clearance from the circulation, and low sensitivity for *Candida krusei* and *Candida parapsilosis* [64,65]. Molecular techniques, including T2 Magnetic Resonance (T2MR), polymerase chain reaction (PCR) assays, and Next Generation Sequencing (NGS) are characterized by high sensitivity and specificity. However, their high cost limits their availability in most settings [23,53,66].

The potential detrimental effects of systemic candidiasis in neonates, in terms of survival and long-term morbidity, and the inability of clinical diagnosis, alongside the significant limitations of the diagnostic techniques described above, lead to the common practice of empirical antifungal administration [53]. Interestingly, Aliaga et al., using data from 322 NICUs, reported an increase in the administration of empirical antifungal treatment rising from 4 to 11.5 per 1000 patients over a 14-year period during which the rate of invasive *Candida* infections declined from 3.6 to 1.4 per 1000 patients [67]. In a large cohort of VLBW neonates, Fortmann et al. reported that more than 95% of antifungal treatment courses were administered empirically [54]. Recently, the CALYPSO study, which was conducted in eight European countries, revealed that 69% of antifungal courses administered in NICUs were empiric [30,68]. The results of the GARPEC study revealed no significant difference in the rate of empirical antifungal treatment in neonates between high income countries and low- and middle-income countries [69]. The challenges associated with timely and accurate diagnosis, leading to the initiation of empirical treatment, are also evident in a pediatric cohort, comprising children beyond the neonatal period. In this group, only 19.7% of children treated with antifungal agents had a proven infection [68].

A further issue regarding empirical antifungal treatment concerns the administration of prolonged courses when *Candida* infection is not eventually proven. Despite the lack of research evidence on prolonged antifungal courses in the absence of proven infection in neonates, insights can be drawn from the extant literature on antimicrobial use, which indicates that antibiotic cessation following negative cultures is associated with decreased exposure and no increase in treatment failure [70,71,72]. A study conducted at our center demonstrated a decrease in LOS episodes and almost complete elimination of invasive *Candida* infections following changes in antibiotic use practices that reduced the duration of antibiotic treatment in suspected and confirmed sepsis cases, and the utilization of narrow-spectrum antibiotics [73].

### 3.2. Challenges with Antifungal Use in Neonates

Evidence on safety, efficacy, and optimal dosing in this population is often limited or inconclusive. The challenge of identifying the optimal dose to achieve adequate exposure primarily can be attributed to the paucity of large pharmacokinetic and pharmacodynamic studies in neonatal populations. Furthermore, the majority of extant studies in neonates are designed to achieve exposure documented as efficacious in adult or pediatric cohorts [74]. In the absence of robust evidence, considerable variation in dosing regimens and treatment durations has been observed across centers, which may lead to over- or underdosing [53,69,75].

A significant variation in the dosage of fluconazole, the most frequently used drug in neonates, is observed in different neonatal cohorts [55]. Subtherapeutic doses that fail to achieve adequate exposure have been reported [53,75]. The multicenter ARPEC study reported that subtherapeutic fluconazole doses were administered in 63% of cases [75]. Due to pharmacokinetic differences in neonates, the administration of a loading dose of 25 mg/kg has been associated with a more rapid attainment of the therapeutic target [39,76]. However, both ESCMID and IDSA state that further evidence is required before recommendations can be made [31,32]. The recently published global guidelines for the diagnosis and management of invasive candidiasis recommend the administration of a loading dose [77]. Therefore, variable practices are adopted in different centers [39].

The broad-spectrum activity of echinocandins, including various non-*albicans Candida* spp., and their activity against biofilms render them useful in the NICU, and their use is continuously expanding [58]. However, the optimal dosage for neonates has not yet been established. Significant variations in the dosage of micafungin, the only echinocandin approved for neonatal use, are observed [31,32]. Pharmacokinetic studies have demonstrated that higher weight-based doses are required in neonates compared to children and adults to achieve adequate exposure and CNS penetration [49]. Current guidelines recommend 4–10 mg/kg/day of micafungin as a salvage treatment in cases of resistance to conventional antifungals [31,77]. Higher doses are needed in cases where CNS involvement is documented or cannot be excluded [78]. Doses up to 15 mg/kg/day have been studied in preterm populations [79]. However, according to the Food and Drug Administration (FDA), doses exceeding 4 mg/kg are not recommended due to insufficient evidence [50]. Caspofungin, although not currently approved for neonatal use, is recommended in cases of resistance to other antifungals at a dose of 25 mg/m^2^/day [31]. Nevertheless, the suggested dosing regimen is based on extrapolation from pediatric and adult populations [80].

A paucity of evidence is highlighted in the current guidelines regarding the optimal duration of antifungal treatment for neonates, with current practices being largely derived from data on other age groups [31]. As Ferreras-Antolin et al. reported, significant variation in the treatment duration of neonates with invasive candidiasis was observed, with a median of 16 (IQR 7–23) days reported [53].

### 3.3. Irrational and Inappropriate Use of Antifungal Prophylaxis

It has been established that the most effective measure to reduce invasive candidiasis among preterm neonates is primary prevention of *Candida* colonization, achieved by reducing exposure to predisposing factors and horizontal transmission [81]. However, the potential detrimental effects of systemic *Candida* infection in this population, and the unavoidable exposure to several predisposing factors during their prolonged NICU stay, have led to the widespread practice of antifungal prophylaxis for neonates at higher risk of infection [82]. Indeed, antifungal prophylaxis represents the most common indication for antifungal administration in the NICU [30]. Fluconazole is currently the most widely prescribed and recommended antifungal drug for prophylaxis in preterm neonates [30,31,32]. A significant decrease in the rates of *Candida* colonization and candidiasis-associated mortality following fluconazole prophylaxis has been reported in VLBW and ELBW populations [83,84,85].

However, the extant evidence is mainly derived from studies conducted in centers with a high prevalence of *Candida* infections [84]. It has been suggested that the benefit from antifungal prophylaxis is probably less significant in settings with a low incidence of invasive candidiasis, as is the case in the majority of NICUs in high-income countries. The current recommendations suggest a risk stratification strategy [31,32]. For ELBW neonates in NICUs with an invasive candidiasis incidence >10%, fluconazole is recommended at 3–6 mg/kg, orally or intravenously, twice weekly for 6 weeks [31] (Table 2). In NICUs with lower candidiasis incidence, fluconazole prophylaxis for ELBW neonates should be individualized based on the presence of additional risk factors, such as the administration of third-generation cephalosporins, prolonged parenteral nutrition, or a central venous catheter [32]. Targeted fluconazole prophylaxis has been shown to effectively reduce invasive candidiasis rates in the NICU [86,87]. Nevertheless, it has been documented that antifungal prophylaxis is frequently administered to neonates who do not fulfill the established criteria. A recent multicenter study revealed that only 34% of neonates who received antifungal prophylaxis were in accordance with the recommendations. Interestingly, only 40.8% of them were ELBW neonates [53]. The CALYPSO study reported comparable observations, with 35% of neonates receiving antifungal prophylaxis being ELBW [30].

According to the current guidelines, a dose of 3 or 6 mg/kg is suggested. A meta-analysis by Leonart et al. reported comparable efficacy between the two doses. Therefore, the authors concluded that it is reasonable to administer the lowest dose to minimize exposure [88]. However, pharmacokinetic studies have indicated that the 3 mg/kg dose may not be efficacious against *Candida* spp. with a minimum inhibitory concentration (MIC) above 2 µg/mL. Consequently, regional *Candida* MICs should be considered when selecting the prophylactic dose [89,90].

The biweekly regimen of fluconazole has been proven to be effective in preventing invasive candidiasis while reducing drug exposure [90,91]. However, a European survey involving NICUs from 28 countries reported that 55% of participating NICUs administered fluconazole prophylaxis biweekly, and 22% and 10% used 48 and 24 h, respectively [92].

Current guidelines weakly recommend oral nystatin at 100,000 IU for antifungal prophylaxis when fluconazole-resistant strains are suspected [31,32,77]. However, data regarding efficacy remains inconclusive, especially for the most vulnerable population with a birthweight of less than 750 g. Moreover, the essential oral route of administration may preclude its use in clinically unstable neonates [31,32,93,94]. Although not currently recommended, micafungin may represent a reasonable choice for *Candida* prophylaxis in settings with high rates of *Candida auris*, which is typically resistant to conventional antifungals used in neonates [95].

## 4. Consequences of Inappropriate Use of Antifungals

### 4.1. Microbiological Consequences

#### 4.1.1. Emergence of Resistance

*Candida* spp. resistance is categorized as intrinsic, which occurs naturally, or secondary, which arises in genetically susceptible strains following exposure to antifungals. Secondary resistance is frequently associated with modified gene expression [96]. It is well-documented that targeted therapy, based on susceptibility testing, the avoidance of subtherapeutic or transiently excessive doses, and the limitation of unnecessary prolonged antifungal administration, has the potential to mitigate the risk of resistance emergence [97,98]. The inappropriate and irrational use of antifungals has significantly contributed to changes in *Candida* epidemiology over the last decade, resulting in an increased prevalence of antifungal-resistant *Candida* species [14,99].

There are significant geographic variations in *Candida* species resistance patterns. In a large Canadian study, including 137 ELBW neonates with invasive candidiasis, *Candida* resistance was uncommon. Fluconazole-resistant strains were isolated in three neonates, and no cases of amphotericin or micafungin resistance were reported [7]. Despite the rarity of resistance to amphotericin B, a Greek retrospective cohort study involving pediatric patients with invasive *Candida* infections over a ten-year period reported a significantly increased median MIC of amphotericin B for *Candida albicans* and *Candida parapsilosis* and of caspofungin for *Candida albicans* [100,101]. Fluconazole-resistant *Candida* species are reported to be prevalent in low- and middle-income countries. In a recent retrospective Turkish study, Ayak et al. reported that 13.2% of *Candida parapsilosis* isolates causing invasive infections were fluconazole-resistant [102]. Moreover, a recent meta-analysis of studies conducted in low- and middle income countries reported a rate of fluconazole-resistant *Candida* parapsilosis of almost 25% [4]. Compared to high-income countries, the overall use of fluconazole is reported to be much higher in low- and middle-income countries [69].

A significant concern arising from the widespread use of fluconazole prophylaxis in the NICUs is the potential emergence of fluconazole-resistant *Candida* strains [103]. The duration of prophylaxis administration, the cumulative dose, the dose interval, and the proportion of admitted patients receiving prophylaxis are factors that could be implicated in the development of resistance [103,104]. Furthermore, prolonged subtherapeutic levels, resulting in drug concentrations below the MIC, have been shown to be associated with the emergence of resistant strains in a murine model [105].

Several studies in neonatal populations have investigated the association between fluconazole prophylaxis and the development of resistance. Luparia et al. reported no increased prevalence of fluconazole-resistant *Candida* spp. during the 16-year period in which routine prophylaxis was implemented. The authors posit that the absence of resistance emergence could be at least partly attributed to the low cumulative fluconazole dose administered, and that the proportion of treated neonates was less than 30% of those admitted in the NICU [104]. Moreover, in several neonatal cohorts, no cases of fluconazole resistance have been reported following fluconazole prophylaxis [106,107,108]. In a retrospective historical-comparative analysis, Zhang et al. reported a statistically significant decrease in the complete fluconazole sensitivity of *Candida* spp. during the period of fluconazole prophylaxis compared to a prior period when no universal prophylaxis for VLBW was implemented (40% vs. 85%, *p* < 0.05) [85]. Another recent study by Autmizguine et al. reported a slight, albeit clinically insignificant, increase in MICs in the group of neonates that received prophylactic fluconazole for periods exceeding one month [103]. Sarvikivi et al. reported the emergence of fluconazole-resistant *Candida parapsilosis* ten years after the implementation of routine prophylaxis. The authors state that these isolates were detected following a period during which prophylaxis was administered to all admitted neonates, rather than being reserved only for high-risk neonates [109]. In a Korean study involving 423 ELBW neonates in two NICUs with a 4.7% incidence of invasive *Candida* infections, a statistically insignificant increase in invasive candidiasis caused by fluconazole-resistant *Candida parapsilosis* was observed following the implementation of fluconazole prophylaxis, compared with a historical control cohort (41.7% vs. 0%, *p* = 0.11) [110].

The irrational and inappropriate use of antifungals contributes to the emergence of intrinsically less susceptible or resistant *Candida* species. A global increase in the prevalence of non-*albicans Candida* species has been documented, with a particularly high incidence observed in low- and middle-income countries [4,14]. A significant concern is the recent emergence of the multidrug-resistant *Candida auris* [111]. The NeoOBS study, which included neonates from eight low- and middle-income countries, reported that *Candida auris* was the third most prevalent species causing invasive infection, accounting for 14% of *Candida* infections [11]. *Candida auris* is characterized by rapid dissemination within hospital environments, prolonged environmental persistence, and rapid nosocomial transmission, thereby contributing to the emergence of endemic nosocomial infections and outbreaks. Horizontal transmission represents the predominant route of *Candida auris* infection acquisition. Lapses in infection control policies and practices have been identified as a primary risk factor for infection and NICU outbreaks [95,111].

#### 4.1.2. Antifungal Tolerance, Persistence, and Outbreaks in the NICU

It is evident that the inappropriate use of antifungals shapes the ecology of *Candida* spp. in the NICU by suppressing susceptible *Candida* populations and selecting for tolerant species capable of persisting on medical devices and in the NICU environment.

The ability of *Candida* species to form biofilms, organized structures surrounded by extracellular matrix, on surfaces and medical devices poses a particular challenge in the treatment of invasive candidiasis in the NICU, as the use of central vascular catheters and medical devices, such as endotracheal tubes, is an integral component of the routine care of preterm and critically ill neonates. Biofilms can both initiate and sustain infections, as pathogens are shielded from the host immune response and antifungal agents [112,113].

Biofilm tolerance to antifungal drugs is associated with the barrier provided by the extracellular matrix to antifungal diffusion, increased metabolic activity during biofilm development, altered gene expression, including upregulation of CDR and MDR genes, and the presence of dormant persister cells, which survive despite antifungal concentrations above MIC [114,115,116]. Moreover, the presence of different fungal and bacterial species within biofilms that interact synergistically or antagonistically is common, further challenging treatment [114].

Healthcare-associated transmission represents the most common route of *Candida* acquisition in the NICU [14]. The ability of several *Candida* species to survive on surfaces and medical devices for extended periods is a significant contributor to nosocomial transmission and outbreaks in NICUs [14,117]. It has been demonstrated that both *Candida parapsilosis* and *Candida auris* have the capacity to persist in the environment for a period exceeding three weeks [118,119]. Moreover, *Candida auris* has been shown to rapidly contaminate surfaces within four hours following disinfection [120]. To avert an outbreak in the NICU, a multidisciplinary approach is imperative to implement strategies that prevent *Candida* dissemination [120].

The combination of environmental persistence and increasing resistance among *Candida* spp. contributes to outbreaks in NICUs. Documented cases of clonal spread of *Candida* spp. through contaminated medical equipment and surfaces, transmitted through health workers’ hands, have been reported in NICUs worldwide, particularly in low- and middle-income countries [14]. Despite documentation of various *Candida* species implicated in NICU outbreaks, there is significant concern about recently reported outbreaks involving antifungal-resistant species, including fluconazole-resistant *Candida parapsilosis* and the multidrug-resistant *Candida auris* [121,122,123,124].

### 4.2. Clinical Consequences

#### 4.2.1. Antifungal Drug Toxicity

The immature renal and hepatic function in neonates renders them particularly susceptible to drug-related toxicities. The pharmacokinetics of drugs are significantly affected by the unpredictable alterations in hepatic clearance and glomerular filtration rate. This poses a considerable challenge in determining optimal dosing [74].

Amphotericin B adverse effects, which mainly include nephrotoxicity, electrolyte disturbances and hepatotoxicity, are less frequently observed in neonates compared to adults [36,56]. Nevertheless, the pharmacokinetics of amphotericin in neonates are characterized by a high degree of variability. This can result in unexpected toxicity, and careful monitoring is therefore required [74,125].

The most frequently documented adverse effects of azoles is hepatotoxicity, characterized by conjugated hyperbilirubinemia and/or elevation of liver enzymes, and gastrointestinal disturbances [33,126]. Due to their immaturity and the presence of other factors that predispose them to liver injury, such as parenteral nutrition, sepsis, and antibiotic administration, neonates, particularly preterm neonates, are prone to liver toxicity [126]. Photosensitivity and visual disturbances have been reported in adult patients receiving voriconazole [18,33]. Phenotypic differences and genetic polymorphisms of the cytochrome P450 2C19 enzyme have been demonstrated to be associated with high inter-individual variability in voriconazole concentrations and difficult-to-predict pharmacokinetics [127].

Elevation of liver enzymes and gastrointestinal irritation are the most frequently reported adverse effects of echinocandins. However, the evidence in the neonatal population is limited [58]. A black box warning has been issued for micafungin due to an elevated risk of hepatocellular tumors observed in experimental animals following prolonged administration [49]. A concern regarding the use of anidulafungin in neonates is the potential for accumulation of polysorbate 80, due to the immaturity of neonates’ detoxification systems [128].

#### 4.2.2. Drug Interactions

Concomitant administration of various drugs is a common practice in the NICU, particularly in critically ill and preterm neonates. It is therefore imperative to consider potential drug interactions to avoid adverse effects and both excessive and subtherapeutic exposure.

The nephrotoxic effects of amphotericin are well-established. The administration of amphotericin concomitantly with other potentially nephrotoxic drugs, including aminoglycosides and vancomycin, should be minimized [129]. Moreover, the co-administration of amphotericin and corticosteroids increases the risk of hypokalemia, necessitating close monitoring [129]. Triazoles are cytochrome P450 inhibitors. Close monitoring or dose adjustment may be necessary when they are administered with other drugs that induce or inhibit cytochrome P450, such as caffeine and midazolam [37,126].

#### 4.2.3. Effects on Mycobiome

The intestinal microbiota consists of a diverse community of microorganisms that coexist in balance. This heterogeneous community, comprising bacteria, fungi, and viruses, plays a crucial role in both early life and long-term health [130,131]. Both genetic and environmental factors influence the composition of the intestinal microbiota [131,132]. The disruption of the microbiota’s balance, intestinal dysbiosis, has been associated with alterations in immune system regulation, intestinal barrier dysfunction, metabolic abnormalities, and inflammation activation [133]. Although present at low abundance in the gastrointestinal tract, fungi are crucial components of the intestinal microbiota. The mycobiome is imperative for intestinal homeostasis, and its alterations have been implicated in the pathogenesis of disease [131,134].

Antifungal agents act against intestinal colonizing fungi, including beneficial species. Although not extensively studied, evidence suggests that excessive or long-term antifungal treatment can disrupt the intestinal mycobiome and microbiota and be implicated in the pathogenesis of diseases [131,135]. Wheeler et al. evaluated the effects of prolonged fluconazole administration in mice, and observed increased severity of colitis in both acute and chronic models of the disease, as well as exacerbation of allergic airway disease. The researchers detected alterations in the microbiota composition, affecting both fungi and bacteria. A decrease in the proportion of *Candida* spp. was observed, accompanied by an increase in *Aspergillus*, *Wallemia,* and *Epicoccum*. The study also observed alterations in the composition of bacterial strains, characterized by a decline in *Bacteroides*, *Clostridium*, and *Lactobacillus* spp., and an increase in *Anaerostipes*, *Coprococcus*, and *Streptococcus* detection [136].

Despite the paucity of data on the correlation between mycobiome alterations due to antifungal exposure during early life and long-term outcomes, the potential implications of mycobiome disruption and the pathogenesis of diseases of inflammatory origin, allergic diseases, and obesity have been demonstrated [135,137,138,139].

A growing body of evidence suggests that the intestinal mycobiome plays a pivotal role in early life, influencing children’s health and development through mechanisms involving immune modulation, metabolic programming, and interactions between bacteria and fungi [140]. The interactions between the developing immune system and the gut mycobiome during infancy have long-term implications for immune health [137]. Atopic diseases have been associated with alterations in the composition of the intestinal mycobiome in early life [137,140]. Current literature indicates that there is a critical period during early life, the first 100 days, the period of greatest plasticity of the immune system, in which alterations in the intestinal microbiota have a greater potential to be implicated in immune dysregulation and the development of atopy and asthma [141]. Fujimura et al. demonstrated that alterations in both the microbiota and the mycobiome are implicated in atopic disease and asthma [142]. Moreover, Arietta et al. demonstrated that the increased abundance of the yeast *Pichia kudriavzevii* at three months of age is associated with an elevated risk of asthma at five years [143].

Recent literature has indicated an association of the intestinal mycobiome with growth during childhood [144]. Gutierrez et al. demonstrated an association between the maturational patterns of the intestinal mycobiome during infancy and the abundance of different fungal strains, including *Saccharomyces*, *Rhodotorula*, and *Malassezia*, with body mass index (BMI) z scores up to the age of five years [145]. In a case–control study, Borgo et al. compared intestinal microbiota diversity between school-aged obese and non-obese children. Although no significant correlation was observed between bacterial biodiversity and obesity, lower fungal abundance was observed, including *Candida* and *Saccharomyces* species [146]. Furthermore, in a prospective cohort of 278 children, higher intestinal fungal abundance at one year was associated with lower BMI, and at two years with higher stature between the ages of two and nine years [144].

In addition to its impact on early childhood, there is a substantial body of evidence supporting the association of mycobiome dysbiosis and morbidities that persist or onset during adulthood. The association between specific fungi and diseases has been demonstrated, as well as a correlation between mycobiome disruption and pathologies affecting various organ systems, including cardiovascular, gastrointestinal, liver, neurological, lung, autoimmune disorders and cancer [147]. Dysbiosis, such as an increase in *Candida* abundance, induces chronic intestinal inflammation through Th17 immune responses and is implicated in the pathogenesis of inflammatory bowel disease [131]. *Candida* Th17 immune responses have also been linked to the pathogenesis of respiratory diseases. The lung-brain axis determines susceptibility to chronic lung disease, and dysbiosis is an independent risk factor for chronic inflammatory lung diseases [137]. The mycobiome disruption is implicated in the onset and progression of diabetes, atherosclerosis, hypertension, and obesity through several mechanisms, including metabolite release, the immune-metabolic axis, and interspecies interactions [148]. Alterations in the intestinal mycobiome have also been associated with neurological disorders, including schizophrenia, Rett syndrome, autism spectrum disorders, and Alzheimer’s disease, suggesting a role for fungi in the gut–brain axis [131,149].

Research into the role of the mycobiome in health and disease is a rapidly evolving field, and knowledge in this area is growing continuously. Future longitudinal studies are required to investigate the association between antifungal-driven alterations in the intestinal mycobiome and the pathogenesis of diseases.

## 5. Antifungal Stewardship Programs in the NICU

Antifungal agents are commonly prescribed drugs in the NICU. However, there is considerable evidence to suggest that these agents are frequently used inappropriately and/or irrationally [53]. To minimize the consequences of antifungal overuse, such as the emergence of resistant strains and increased toxicity risks, strategies should be implemented in the NICUs to optimize antifungal use. The development of antifungal stewardship programs (AFSs) within NICUs is imperative to address the specific challenges posed by this population [55,150].

### 5.1. Targets of AFS in the NICU

The implementation of AFS in the NICU is intended to facilitate optimal utilization of antifungals, considering the distinctive characteristics and challenges of this population [55]. This approach is expected to enhance clinical outcomes and mitigate the risks associated with inappropriate use.

A major barrier to optimizing antifungal use is the limitations of diagnostic methods and the lack of rapid, accurate diagnostic techniques in most settings. Therefore, *Candida* infections may be both overtreated and underdiagnosed. A primary objective of AFS is to reduce empirical treatment and instead promote targeted treatment [150,151]. Adequate blood volume should be obtained to enhance the detectability of *Candida* species in blood cultures. This is however particularly challenging in preterm and critically ill neonates [60]. Although there is limited evidence in neonatal populations, serum biomarkers, such as mannan/anti-mannan antibodies and 1,3-β-D-glucan could potentially be useful in the NICU due to their high sensitivity and negative predictive value [62,65]. However, the implementation of molecular techniques for *Candida* diagnosis, including PCR-based technologies, spectroscopy-based methods, and sequencing, in daily clinical practice is a crucial step to ensure rapid, accurate diagnosis. These techniques have the potential to facilitate targeted treatment by providing accurate identification of *Candida* species and antifungal susceptibility testing. However, the high cost of these techniques significantly restricts their widespread utilization [23,66].

Nevertheless, despite the efforts to restrict empirical treatment when invasive *Candida* infections are suspected, it is often inevitable, given the nonspecific clinical presentation and the potential detrimental effects of untreated infections. The objective of AFSs involves the optimization of empirical treatment when necessary [151]. The choice of the antifungal agent should be based on local susceptibility *Candida* patterns.

Although there is a considerable lack of clarity regarding the appropriate dosing of antifungals and the duration of treatment in neonates, AFSs should aim to ensure that these are in accordance with existing guidelines. Once susceptibility of *Candida* spp. has been determined, clinicians should be encouraged to de-escalate treatment [55].

In addition to the anticipated microbiological and clinical advantages of implementing AFS, a documented reduction in economic burden has been observed. Although evidence on the implementation of AFS in NICUs is limited, data from pediatric and adult settings have demonstrated cost reductions [150,152,153]. Soni et al. reported a 59.9% reduction in antifungal costs over a 5-year period following of AFS implementation [150].

A significant component of AFS in the NICU is optimizing antifungal prophylaxis for high-risk neonates. Caution should be given to ensure adherence to the current guidelines and the development of a local protocol with clear indications for prophylaxis administration [55]. It has been demonstrated that effective antifungal prophylaxis is associated with a considerable reduction in the economic burden of antifungals in NICUs [154,155]. Uko et al. estimated that the financial burden was reduced by $663,638 following the implementation of a targeted fluconazole prophylaxis strategy over an 18-month period [155]. Furthermore, financial savings have been demonstrated through reduced need for diagnostic examinations to evaluate disease dissemination, prolonged hospitalization, and reduced need for further hospitalization due to the disease’s long-term sequelae [156].

### 5.2. Implementation of AFS in the NICU

The effective implementation of an AFS program in a NICU requires a systematic approach that incorporates meticulous planning and organizational structure. The core elements of an AFS comprise a multidisciplinary team approach, microbiological surveillance, diagnostic optimization, the establishment of guidance for optimal use of prophylaxis and treatment, and regular audits to ensure and/or enhance compliance [55,150,151,157].

**Figure 1:**
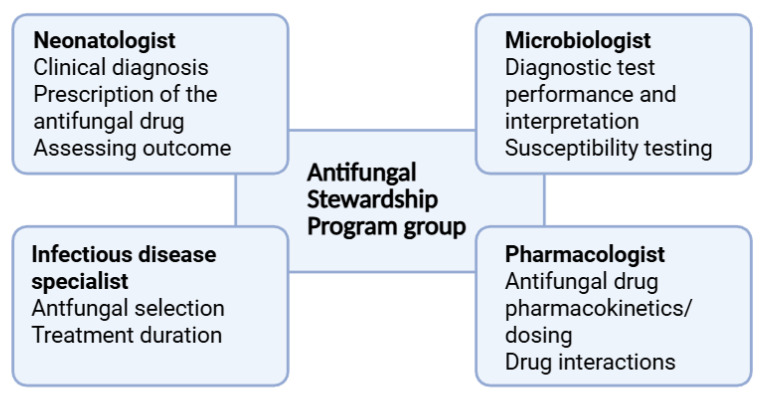
The multidisciplinary approach in the Antifungal Stewardship Program.

It is crucial to acknowledge the necessity of a multidisciplinary approach, given the expertise required of AFS team members in areas such as clinical courses of invasive *Candida* infections, the epidemiology and susceptibility patterns of *Candida* spp., laboratory techniques, pharmacokinetics of antifungal agents, optimal dose and duration, drug toxicities, and interactions [157,158]. This group of experts should include neonatologists, infectious disease specialists, clinical pharmacologists, and microbiologists (Figure 1). Regular meetings of the AFS team to both organize the strategy and assess the associated results, together with collaboration with the attending clinicians in the NICU, should be considered a priority [150,157,158].

Efforts should be made to optimize laboratory diagnosis and specification of *Candida* strains, to guide targeted treatment. Close collaboration with the microbiology department is imperative in order to maximize the utilization of the available diagnostic techniques, to reliably evaluate test results, and expand the use of molecular technologies [150,151,157].

**Figure 2:**
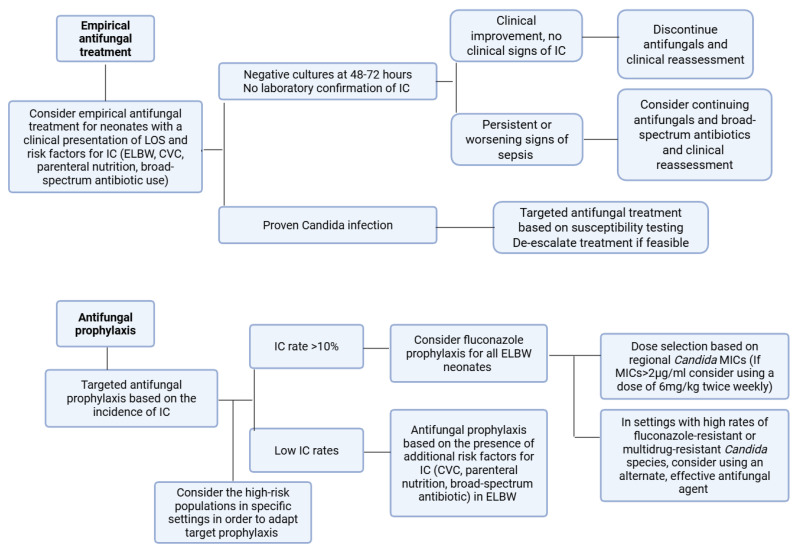
Proposed flowchart for antifungal prophylaxis and treatment of neonatal fungal infections. IC: invasive candidiasis; ELBW: extremely low birthweight; CVC: central venous catheter; LOS: late-onset sepsis; MIC: minimum inhibitory concentration.

Predefined criteria regarding the cessation of treatment in cases where *Candida* infection is not ultimately confirmed should be established. The development of algorithms could offer clinicians a valuable tool to prevent unnecessary prolonged treatment courses (Figure 2) [55]. In addition to the existing guidelines, local epidemiology and *Candida* resistance patterns should be incorporated to form a local protocol for the management of invasive candidiasis. This protocol should include recommendations on the choice of antifungal agent, dosing, treatment duration, and de-escalation of treatment when possible [151,157,159].

Electronic prescription, in addition to minimizing errors and protocol deviations, are an efficient way to monitor adherence. The created databases can be used for audits, which are necessary to provide clinicians with feedback, evaluate AFS outcomes, and set objectives [159].

## 6. Future Perspectives

The implementation of AFSs has the potential to mitigate the inappropriate and irrational use of antifungals in NICUs, thereby preventing the clinical and microbiological consequences arising from misuse. Nevertheless, there is a paucity of well-designed studies evaluating the efficacy of AFS in the NICUs. It is therefore reasonable to hypothesize that future studies will demonstrate the benefits of this strategy and encourage more centers to implement it. The establishment of a collaborative relationship between a network of institutions on an international level could facilitate the exchange of experiences from different centers, the exchange of ideas, and the attraction of further centers to implement AFS.

However, optimization of antifungal use in neonates necessitates the determination of the optimal dose of antifungals for both term and preterm neonates. Pharmacokinetic and pharmacodynamic studies in the neonatal population are necessary to identify the safe and effective dose of each antifungal agent, thus providing the basis of international guidelines for all agents. Furthermore, research could address the validation of diagnostic techniques, particularly serum biomarkers, for application in the neonatal population. Serum biomarkers are low-cost techniques that could be utilized in most settings and, therefore, be useful tools in *Candida* diagnosis.

## 7. Conclusions

Neonatal invasive *Candida* infections are a major global concern, as they are associated with significant morbidity and mortality. Prophylaxis with antifungal drugs has been demonstrated as an efficient strategy to reduce the burden of the disease in high-risk infants. However, administration of prophylaxis in neonates outside the recommended indication is frequently documented. Moreover, the unspecific clinical presentation, the limitations of diagnostic techniques, and the lack of robust evidence on optimal dosing and treatment duration often result in the inappropriate and/or irrational use of antifungals. Misuse of antifungal agents has been demonstrated to be a significant contributing factor to the increasing prevalence of non-*albicans Candida* strains, which are intrinsically less susceptible, and the emergence of antifungal-resistant strains. Moreover, clinical consequences arising from antifungal misuse, including drug toxicities and interactions and effects on the intestinal mycobiome, have been well documented.

It is therefore apparent that there is an urgent need for the optimization of antifungal drug use in neonates. The implementation of AFSs is a promising strategy to optimize antifungal use in NICUs. The multidisciplinary team approach, the development of protocols according to the local epidemiology and resistance patterns, algorithms to inform clinicians on treatment initiation and cessation, and audits and feedback are the core of AFS. However, further research into neonatal populations is essential to improve *Candida* diagnosis and determine the optimal antifungal dosage and treatment duration for both term and preterm neonates.

## Figures and Tables

**Table 2:** Summary of current guidelines on antifungal prophylaxis in neonates [31,32].

**ESCMID 2012**
Fluconazole prophylaxis 3–6 mg/kg iv or per os biweekly. For all ELBW neonates in NICUs with a high incidence of IC (*strong recommendation; high-quality evidence*) Targeted prophylaxis using a risk stratification strategy (ELBW neonates with additional risk factors for IC, such as CVC, broad-spectrum antibiotics, and prolonged parenteral nutrition) in NICUs with low incidence of IC (*moderate recommendation; moderate-quality evidence*)
Lactoferrin 100 mg/day alone or in combination with Lactobacillus 10^6^ colony-forming units once daily (*moderate recommendation; moderate-quality evidence*)
Nystatin 100,000 U per os every 8 h *(moderate recommendation; moderate-quality evidence)*
**IDSA 2016**
Fluconazole prophylaxis 3–6 mg/kg iv or per os biweekly for six weeks for all ELBW neonates in NICUs with a high incidence of IC (>10%) (*strong recommendation; high-quality evidence*)
Lactoferrin 100 mg/day per os (*weak recommendation; moderate-quality evidence*)
Nystatin 100,000 U per os every 8 h for six weeks, in cases where fluconazole cannot be used due to unavailability or resistance (*weak recommendation; moderate-quality evidence*)

ESCMID: European Society of Clinical Microbiology and Infectious Diseases; IDSA: Infectious Diseases of North America; iv: intravenous; NICU: neonatal intensive care unit; IC: invasive candidiasis; ELBW: extremely low birth weight; CVC: central venous catheter.

## Data Availability

No new data were created or analyzed in this study. Data sharing is not applicable to this article.
